# Identification of the source of biliary bleeding using a novel ultrathin‐type peroral cholangioscope

**DOI:** 10.1111/den.15061

**Published:** 2025-06-11

**Authors:** Kento Shionoya, Ryosuke Tonozuka, Takao Itoi

**Affiliations:** ^1^ Department Gastroenterology and Hepatology Tokyo Medical University Tokyo Japan

## Abstract

Watch a video of this article.

## BRIEF EXPLANATION

A peroral cholangioscope (POCS) allows direct observation of the inside of the bile duct and is widely used in diagnosing and treating biliary tract lesions.^1^, ^2^ In recent years, various types of biliary lesion classification systems have been developed and clinically applied.^3^ This video presents a case in which a novel POCS was useful for detecting the source of biliary bleeding.

**Figure 1:**
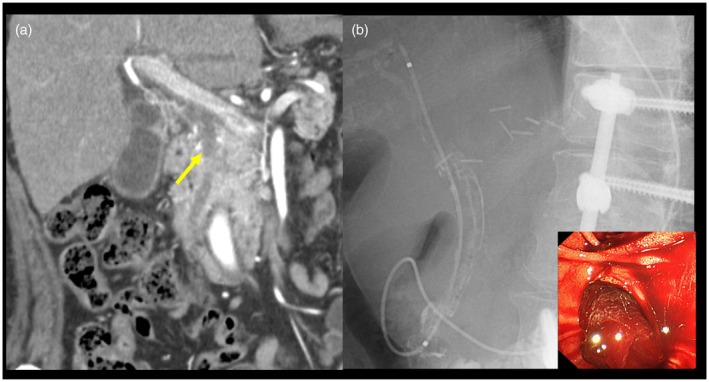
Image findings on admission. (a) Computed tomography revealed a high absorption area from the hilar to the distal bile duct (arrow). (b) Emergency endoscopic retrograde cholangiopancreatography was performed. Although profuse bleeding was observed from the bile ducts, no obvious bleeding source was identified. An endoscopic nasobiliary drainage tube was deployed.

**Figure 2:**
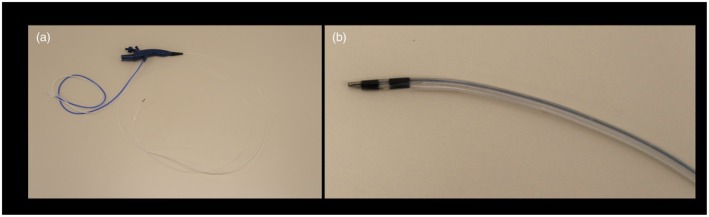
A novel ultrathin‐type peroral cholangioscope. (a,b) A novel ultrathin‐type peroral cholangioscope with an external tip external diameter of 2.3 mm (DRES Slim Scope; Japan Lifeline, Tokyo, Japan).

A 70‐year‐old female patient with cirrhosis of nonalcoholic fatty liver disease and a previous partial hepatectomy for hepatocellular carcinoma (HCC) presented to our institution with abdominal pain and black stools. Computed tomography revealed a high‐absorption area from the hilar to the distal bile duct (Fig. 1a). The patient was diagnosed with acute cholangitis secondary to biliary bleeding. Emergency endoscopic retrograde cholangiopancreatography (ERCP) was performed. Although profuse bleeding was observed in the bile ducts, no obvious bleeding source was identified. An endoscopic nasobiliary drainage (ENBD) tube was deployed (Fig. 1b). Initially, bloody bile drained via the ENBD tube; however, the bloody component gradually decreased. After the acute cholangitis improved, 4 days later, another ERCP was performed to identify the bleeding source. However, the ERCP showed the common and intrahepatic bile ducts were diffusely narrow, and conventional POCS insertion was considered difficult; hence, a novel ultrathin‐type POCS (UTPOCS) with an external tip diameter of 2.3 mm (DRES Slim Scope; Japan Lifeline, Tokyo, Japan) (Fig. 2) was inserted. When the UTPOCS was inserted into B3, biliary bleeding was observed and an ENBD tube was inserted at the site (Video S1). Angiography was performed to achieve hemostasis. It also showed a liver mass in S4. The patient was diagnosed with postoperative HCC recurrence and bleeding from bile duct infiltration, which was successfully treated with embolization. The UTPOCS is useful for direct observation, even inside a narrow bile duct.

Authors declare no conflict of interest for this article.

.

## Supporting information


**Video S1** Identification of the source of biliary bleeding using a novel ultrathin‐type peroral cholangioscope.

## References

[1] Tonozuka R , Itoi T , Nagai K *et al*. A novel peroral digital cholangioscope with a large accessory channel: An experimental study. J Hepatobiliary Pancreat Sci 2023; 30: 401–407. 36043228 10.1002/jhbp.1231

[2] Sakamoto Y , Takeda Y , Seki Y *et al*. The usefulness of peroral cholangioscopy for intrahepatic stones. Clin Med 2022; 11: 6425. 10.3390/jcm11216425 PMC9654474 36362652

[3] Sethi A , Tyberg A , Slivka A *et al*. Digital single‐operator cholangioscopy (DSOC) improves interobserver agreement (IOA) and accuracy for evaluation of indeterminate biliary strictures: The Monaco classification. J Clin Gastroenterol 2022; 56: e94–e97. 32040050 10.1097/MCG.0000000000001321

